# Acellular dermal matrix as filler in breast-conserving surgery: warnings for a careful use

**DOI:** 10.1186/s12957-020-02109-x

**Published:** 2021-01-02

**Authors:** Gianluca Franceschini, Riccardo Masetti

**Affiliations:** Multidisciplinary Breast Unit, Department of Woman and Child Health and Public Health, Fondazione Policlinico Universitario Agostino Gemelli IRCCS, Università Cattolica del Sacro Cuore, Largo Agostino Gemelli, 8-00168 Rome, Italy

**Keywords:** Breast cancer, Breast-conserving surgery, Acellular dermal matrix, Reconstructive biomaterial

## Abstract

Acellular dermal matrices are biological materials of porcine, bovine, or human origin used as scaffold for reconstructive purpose in plastic surgery; these materials are well-tolerated and safely integrated in host tissues without causing resorption, contracture, and encapsulation thanks to their low antigenicity.

Recently, human acellular dermal matrix has been used as a filler in breast-conserving surgery to improve aesthetic results. Adequate knowledge of biomaterials properties, appropriate skill, and careful compliance with some specific recommendations are mandatory in order to optimize outcomes and obtain a work of success.

We read with great interest the original article of Gwak et al. [1] and would like to make some appraisals in order to optimize the use of acellular dermal matrix (ADM) in breast-conserving surgery (BCS).

ADMs are well-known biologic scaffolds of human, bovine, or porcine origin that are increasingly used in field of plastic surgery mainly in breast reconstruction after conservative mastectomy in order to support implants [2]; ADMs are safely integrated in host tissues without causing resorption, contracture, and encapsulation thanks to their low antigenicity [2].

Recently, ADM combined with oxidized regenerated cellulose was also used as aid to obtain better cosmetic results in partial breast reconstruction [3].

Gwak et al. are the first to report improved aesthetic outcomes using only human ADM as a filler in the BCS of 117 breast cancer patients treated at the Division of Breast and Thyroid Surgical Oncology of St. Vincent’s Hospital (Seoul, Republic of Korea) [1].

The evaluation of cosmetic outcomes, 6 months after surgery using a scoring system (0–10 points), documented very positive results, with an average score for overall satisfaction of 9.4 in the patient group and 9.5 in the surgeon group [1].

However, as ADM could be increasingly utilized in breast-conserving surgery, it is crucial to know not only its potential aesthetic benefits but also its possible issues.

Gwak and colleagues reported a 5.1% rate of hematoma, 6.0% of seroma, and 2.5% of red breast syndrome in the 117 breast cancer patients who underwent BCS with ADM; seroma and hematoma accounted for 54.2% of all their complications. In addition, they experienced a significant overall reoperation rate due to complications in 8.5% of their patients; two cases required ADM excision due to hematoma and inflammation [1].

Higher seroma and infections rates were also showed using the coverage of implant with ADM in breast reconstruction after mastectomy [2].

Similarly to other biomaterials, alloplastic ADMs may suffer from poor tissue adherence and may cause allergic skin reactions and seroma as consequence of redundant ADM digestion and a foreign-body reaction with risk of extrusion due to its not optimal bioabsorption [4].

These possible surgical complications could compromise aesthetic outcomes and patient quality of life and at the same time cause a delay in the beginning of adjuvant therapies.

In order to minimize surgical complications and increase the chances of success when ADM is used in BCS, appropriate skills and repetitive practice of standardized tasks may be helpful:

- Careful patient assessment to select the best candidates to BCS with ADM; patients with specific medical comorbidities, active smoking, non-controlled diabetes mellitus or treated with neoadjuvant chemotherapy should not be considered for use of ADM as a filler due to higher risk of postoperative infections.
- Appropriate planning of skin incision that should be performed as far away from the tumour site as possible in order to prevent ADM being directly below surgical suture and minimize the risk of ADM extrusion.
- Meticulous dissection of mammary gland in subdermal fascial plane preserving an adequate subcutaneous thickness in order to ensure an adequate and safe coverage.
- Accurate weight control of removed tissue to determine appropriate reconstructive volumes and carefully calibrate the amount of human ADM to be used as a filler; diced ADM pieces must properly fill the cavity but should not produce swelling of the breast in order to avoid a foreign-body reaction.
- Two glandular flaps should be created by dissection of the residual parenchyma from subcutaneous plane and then sutured together in order to provide an appropriate coverage of ADM-filled cavity.
- Antibiotic therapy could be prophylactically given for at least 5 days in the postoperative period to prevent infections.
- Treatment of postoperative complications should begin as soon as possible with steroids and antihistamine medications in case of red breast syndrome and with repeated percutaneous aspirations in case of seroma to quickly solve the problem and to avoid delay in adjuvant therapies.

Moreover, fibrogenetic action induced by diced ADM and its partial reabsorption can determine peculiar radiological findings on postoperative imaging; in the study of Gwak et al., mammography showed ADM as a mass with well-circumscribed margins [1]; ultrasonography revealed that diced ADM had the same echogenicity as fibroglandular parenchyma presenting a peculiar image similar to that reported using oxidized regenerated cellulose as a filler in BCS [1, 5, 6]; a rim-enhancing mass was found around the ADM on contrast-enhanced MRI that needed core needle biopsy in order to obtain a detailed diagnosis [1]. These peculiar radiological findings can lead to undue alarmism and misdiagnosis during the follow-up of breast cancer patients if not properly interpreted.

In conclusion, the application of ADM could be a feasible option in order to improve aesthetic outcomes in BCS as long as specific and standardized recommendations are followed by surgeons; however, when using ADM as a filler, it is mandatory to inform the patient about its potential aesthetic benefits but also its possible postoperative complications with a detailed informed consent. It is also helpful that breast surgeons describe specifically the use of ADM in the report of the surgical procedure, so that radiologists can correctly interpret the peculiar imaging due to this biomaterial and avoid alarmism and misdiagnosis during the follow-up.

## Data Availability

“Not applicable”

## Abbreviations

ADM: Acellular dermal matrix
BCS: Breast-conserving surgery
